# Genetic Transformation in *Cryptococcus* Species

**DOI:** 10.3390/jof7010056

**Published:** 2021-01-15

**Authors:** Ping Wang

**Affiliations:** Department of Microbiology, Immunology, and Parasitology, Louisiana State University Health Sciences Center, New Orleans, LA 70112, USA; pwang@lsuhsc.edu

**Keywords:** *Cryptococcus*, selectable gene marker, electroporation, biolistic transformation, CRISPR-Cas9 technique

## Abstract

Genetic transformation plays an imperative role in our understanding of the biology in unicellular yeasts and filamentous fungi, such as *Saccharomyces cerevisiae, Aspergillus nidulans, Cryphonectria parasitica*, and *Magnaporthe oryzae*. It also helps to understand the virulence and drug resistance mechanisms of the pathogenic fungus *Cryptococcus* that causes cryptococcosis in health and immunocompromised individuals. Since the first attempt at DNA transformation in this fungus by Edman in 1992, various methods and techniques have been developed to introduce DNA into this organism and improve the efficiency of homology-mediated gene disruption. There have been many excellent summaries or reviews covering the subject. Here we highlight some of the significant achievements and additional refinements in the genetic transformation of *Cryptococcus* species.

## 1. Introduction

Genetic transformation plays an imperative role in our understanding of the biology in unicellular yeasts and filamentous fungi, including *Saccharomyces cerevisiae*, *Aspergillus nidulans*, *Cryphonectria parasitica*, and *Magnaporthe oryzae*. It also helps to understand the virulence and drug resistance mechanisms of the pathogenic fungus *Cryptococcus* that causes cryptococcosis in health and immunocompromised individuals. Since the first attempt at DNA transformation in this fungus by Edman in 1992, various methods and techniques have been developed to introduce DNA into this organism and improve the efficiency of homology-mediated gene disruption. There have been many excellent summaries or reviews covering the subject. Here we highlight the major technical achievements in the genetic transformation of *Cryptococcus* species and additional refinements to increase gene editing efficiency in the fungus.

## 2. Genetic Elements Required for Transformation

Transformation is a process where foreign materials such as DNA are introduced into an organism so that the genetic information can be expressed. For fungi, the first successful attempt was made by Hinnen and Beggs in the 1970s when the *LEU2* gene was introduced into a *leu2* auxotrophic mutant of *S. cerevisiae* [1,2]. Under ordinary conditions, transformation results in either the introduced DNA being maintained episomally as a transient “mini” chromosome or stably integrated into the genome through non-homologous end joining (NHEJ) or homology-directed repair (HDR). In either case, the result is the modification of the genotypes resulting in altered phenotypic characteristics. Genetic transformation often requires the following specific elements.

### 2.1. Origin of Replication (ORI) and Autonomously Replicating Sequences (ARSs)

For stable maintenance of the introduced DNA, the replication origin or sequences that resemble the centromeres are usually necessary. In *S. cerevisiae*, the Origin of Replication (ORI) sequence of the 2-micron plasmid is one such example [3]. Another example is the ARS sequence identified from the maize fungal pathogen *Ustilago maydis* [4].

For *Cryptococcus*, a DNA fragment was described by Varma and colleagues to confer the episomal maintenance of DNA as it exhibits an ARS-like function by enhancing the episomal maintenance of the DNA plasmids following transformation [5,6]. A later report, however, questioned the presence of such a sequence [7].

### 2.2. Telomeres

Although no functional replication origins have been identified in *Cryptococcus,* telomeric sequences were identified that presumably increases the stability of the introduced DNA. These sequences were found in unstable transformants of *C. deneoformans* (formerly *C*. *neoformans var. neoformans*) [8]. The utility of these telomere sequences has not been further explored since subsequent research focused largely on integrative transformation.

### 2.3. Promoter and Terminator Sequences

One of the principal utilities for DNA transformation is to express a gene of interest to characterize its function. For this purpose, DNA sequences possessing promoter activities are required to allow constitutive or inducible gene expression. In addition, terminator sequences are also necessary to increase expression efficiency. Endogenous or heterologous genes encoding conserved glyceraldehyde-3-phosphate dehydrogenase (*GPD*) and actin (*ACT*) are often used [9,10,11,12]. The constitutively activated endogenous Histone H3 promoter was also used in cryptococcal research [13,14].

For regulated expressions, the most commonly used promoter is *GAL7*, which is inducible when galactose is used as the sole carbon source [15]. The activity of *GAL7* is also subject to glucose repression, and its utilities have been well characterized in several studies [15,16,17]. In addition to *GAL7*, *GAL1* and *UGE2* promoters were found to be subject to galactose regulation [18]. A copper-inducible promotor, *CRT4,* was also described [9]. Finally, commonly used terminator sequences include those of the tryptophan biosynthesis protein (*TRP1*) and imidazoleglycerol phosphate dehydratase (*HIS3*) genes [19].

### 2.4. Selectable Marker Genes

#### 2.4.1. Auxotrophic Gene Markers

Selectable genetic markers are a prerequisite for any transformation since they provide a means to monitor and select for transformants. The first gene pursued as a marker for cryptococcal transformation is the *URA5* gene that encodes the orotate phosphoribosyltransferase [20]. The advantage of this marker is that *ura*- auxotrophic mutants serving as the DNA recipients can spontaneously occur or be enriched by ethyl methanesulfonate (EMS) and ultraviolet (UV) mutagenesis. The *ura*- mutant strains are also easily identifiable by their resistance to 5-fluoroorotic acid (5-FOA) supplemented in the selection medium. The *URA5* marker has been widely used in studies of *Cryptococcus* [21,22].

The second auxotrophic marker gene is *ADE2* that encodes a phosphoribosyl aminoimidazole carboxylase in the purine biosynthetic pathway of *C. neoformans* (formerly *C. neoformans var. grubii*) [23]. The *ade2* mutants can be distinguished from wild type cells by their pink appearance due to the accumulation of phosphoribosyl aminoimidazole. *C. neoformans* mutant strains used as the DNA recipients in transformation include MO49 (from γ irradiation) and MOO1 (an *ade2* point mutation mutant) derived from the archetypical parental strain H99 [23,24].

In addition to *URA5* and *ADE2*, the *AMDS* gene encoding the acetamidase and the *NMT487D* gene encoding the myristoyl-CoA:protein N-myristoyl transferase were used as selection markers in the transformation of *Cryptococcus* [25,26]. Moreover, a mutation in the gene encoding the 60S ribosomal protein L41 resulting in increased sensitivity to cycloheximide was also explored as a selectable marker [6].

#### 2.4.2. Positive Selectable Gene Marker

While the auxotrophic markers described above are easy to obtain and serve well as convenient markers in transformation, they are nutrient-based, and the mutant stains often exhibit various defects in growth and development. The complemented strains may also differ from the wild type, particularly in virulence, due to varied expression levels of the reintroduced markers. Such a defect would adversely affect the utility of the auxotrophic markers in the examination of gene functions. To overcome this limitation and increase the number of genetic markers, dominant marker genes encoding drug resistance proteins were adopted and introduced into the cryptococcal field. One such maker is the bacterial hygromycin B phosphotransferase (*HPH*) gene for hygromycin B resistance [27].

To reduce the background associated with hygromycin B resistance and to increase selection efficiency, the bacterial *NAT* gene encoding nourseothricin N-acetyl transferase was adopted by McDade and colleagues for cryptococcal transformation [19]. G418 and phleomycin resistance was also described by Hua and colleagues as the selectable markers [28]. Nourseothricin and G418 resistance has become the most frequently utilized genetic markers in cryptococcal research by many research groups, including ours [12,16,29,30].

## 3. Transformation Methods

Unlike *S. cerevisiae* that is amendable to various transformation methods, including heat shock in the presence of lithium acetate and polyethylene glycol [31], transformation for most other fungi, including *Cryptococcus* species, require more elaborate schemes and often costly equipment. Nevertheless, transformation is no longer a limiting factor in cryptococcal research, as several methods are available. Various improvements have also been made to increase transformation efficiency. We here summarize these methods.

### 3.1. Protoplast/PEG-CaCl2

Transformation of protoplasts mediated by polyethylene glycol (PEG) and CaCl2 was the original and standard method for most filamentous fungi or yeasts in which protoplasts can be readily generated. Indeed, it has been the primary approach for several fungal models that we have worked with [32,33, 34]. However, this methodology has never been utilized for *Cryptococcus* studies, despite earlier studies describing the generation of protoplasts and their usages [35,36]. The cryptococcal polysaccharide capsule presents a significant challenge to generating protoplasts.

### 3.2. Electroporation

Electroporation is a widely used method to introduce DNA and other materials into cells via brief electric pulses, which induce transient and reversible cell membrane permeabilization. Electroporation was first utilized for introducing the *URA5* marker gene into *C. deneoformans* by Edman and colleagues in 1990 that established the first transformation example of this organism [20]. Due to the observation that electroporation often results in the presence of episomally maintained DNA that rarely integrates into the chromosomes of the cell. Efforts, including the use of the dominant selection markers, the split marker genes, and, recently, the clustered regularly interspaced short palindromic repeats (CRISPR)-CRISPR-associated protein 9 (Cas9) technique, were made to increase chromosomal integration efficiency and induce HDR [37,38,39,40,41]. We will discuss these efforts later in the chapter.

Typical electroporation of *Cryptococcus* species requires an electroporation system. One of the popular electroporation systems is a modular Gene Pulser Xcell system (Bio-Rad Laboratories, Hercules, CA, USA). No report is seen yet describing the use of the BTX (Biochrom Ltd., Rehovot, Israel) system for cryptococcal research, even though it is capable of handling multi-well electroporation simultaneously. In electroporation, exponentially-growing cells are preferred in either Tris buffer or an electroporation buffer (10 mM Tris-HCl, 1 mM MgCl2, 270 mM sucrose, pH 7.5) that improves efficiency [37,42]. Dithiothreitol (DTT) may also be used to increase the competency of the cells [37]. Electroporation conditions vary depending on the specific electroporator used. For the Bio-Rad Gene Pulser Xcell system with 2 mm gap cuvettes, either the exponential (0.45–0.47 kV, 50–125 µF, and 500 Ω) or the time constant protocol (1.8 to 2.0 kV, 5 ms) can be used [40]. Salt contamination needs to be minimized to avoid arcing, however.

### 3.3. Biolistic Transformation

Biolistic transformation relies on the bombardment of DNA-coated gold or tungsten particles to deliver DNA inside the cells. Once inside the nucleus, DNA could become transiently expressed or, most often, stably integrates into the host genome. Biolistic transformation offers the advantage that the method is versatile and straightforward, requiring no specialized vectors, such as binary vectors. Further, the cells can be co-transformed with multiple plasmids. Biolistic transformation of *Cryptococcus* was first described by Toffaletti and colleagues in 1993 [43]. Despite its high cost in initial equipment investment and costly consumables, the biolistic transformation has been the primary approach for generating gene-specific mutant strains and for insertional mutagenesis studies [44,45,46]. We have used this approach to have characterized a variety of important genes in both *C. neoformans* and *C. deneoformans* [12,29,47, 48, 49, 50].

The Bio-Rad PDS-1000/He particle delivery system (Bio-Rad Laboratories, Hercules, CA, USA) remains the only available option for biolistic transformation. Many detailed steps, as well as adjustments to the biolistic procedure, have been described [51]. Due to the many variables involved, no biolistic transformation efficiency is duplicable. Cell conditions (growth phase and density), DNA (amount and purity, circular or linearized), DNA coating on beads and washing processes, bead coverage, and dehydration degree on the macrocarrier membrane, and vacuum settings, as well as recovery condition and time, are all factors affecting transformation efficiency. For most applications, 0.6 μm microcarriers (gold beads), 1350 psi rupture disc, and a ≥27 psi vacuum are the typical settings. Either ethyl alcohol or isopropyl alcohol can be used to reduce the static charge of the microcarrier membrane and rupture disc. A single plate can be bombarded multiple times to increase the transformant yield. Should low efficiency remain following optimization of all variables, the vacuum pump and tubing connection need to be examined. Indeed, we have experienced a persistent low transformation efficiency due to low vacuum pressure caused by a loose-fitting gas acceleration tube. Wrapping with a thin layer of Teflon tape and refitting remedied the issue for us.

### 3.4. Agrobacterium Tumefaciens-Mediated Transformation (ATMT)

*Agrobacterium tumefaciens* is a plant bacterial pathogen that can insert a piece of DNA into the host cell. This property has been developed into a tool utilized in the transformation of a variety of organisms, including fungi. The most notable utility of ATMT is to generate random T-DNA insertional mutants since this approach does not induce HDR.

The introduction of ATMT for cryptococcal research was first made by McClelland and colleagues in which they reported that ATMT induces several mutations in the capsule genes of *Cryptococcus* [52]. At the same time, Idnurm and colleagues reported that they were successful in utilizing the technique for an insertion mutagenesis study [34]. Because ATMT is generally considered to be very laborious and time-consuming [53,54], in addition to its inability to induce HDR, its usage in cryptococcal research remains limited.

### 3.5. Other Transformation Approaches

In addition to protoplast/PEG-CaCl2, electroporation, biolistic transformation, and ATMT, the liposome-mediated transformation method has also been described for fungal transformation. This method utilizes the unique properties of liposomes being able to undergo cell membrane entrance, endosomal escape, and nuclear uptake processes. The liposome-mediated transformation has been utilized in the transformation of the oyster mushroom fungus *Pleurotus ostreatus* and the black bread mold *Rhizopus stolonifer* (*nigricans*) [55,56]. Since this method is generally reserved for cells lacking a cell wall, its potential application in cryptococcal studies remains questionable. Table 1 summarizes the current transformation methods.

## 4. Enhancing Homology-Directed Repairs (HDRs)

Previous studies revealed that the introduction of DNA into the cell via electroporation rarely induces chromosome integration, resulting in few gene disruption events. Even for biolistic transformation, its efficiency of inducing HDR is also highly varied, ranging from 1–2% to 10–20% [21,51]. Consequently, there have been many efforts made to increase HDR efficiency, including the use of linearized DNA, split-marker genes, Ku70/80 mutant recipients, and Ku protein inhibitors.

### 4.1. Circular vs. Linearized DNA Plasmids

In studies of other fungi, an approach called Restriction-Enzyme-Mediated Integration (REMI) was often used for increasing chromosomal integration. REMI incorporates restriction enzymes during transformation that linearizes DNA to stimulates its integration into partially digested cognate restriction sites in the genome [57]. This approach has not been reported to be used in the cryptococcal transformation. Following findings that electroporation induces unstable extra-chromosomally maintained DNA, HDR studies commonly employ biolistics for transformation. We commonly use linearized plasmids for HDR-mediated gene knockouts and for mutant complementation.

### 4.2. Split Marker Genes

To reduce NHEJ during transformation intended for gene knockouts, the split-marker gene approach has often been employed. This method uses two PCR-amplified fragments, each containing one-half of the marker gene, and transformants that have undergone HDR of both the marker and target genes are positively selected. This process significantly reduces NHEJ frequency [58,59,60] and allows the construction of various specific mutant libraries [61,62].

### 4.3. Additional HDR Enhancing Methods

Following the general practice of including homologous sequences flanking the marker genes to promote HDR, a detailed study demonstrated that the minimal length of homologous sequences should not be less than 300 bp [63]. It was also found that when the Ku-defective mutant strains were used as the recipients, HDR-mediated gene knockout efficiency was dramatically enhanced [64]. The Ku protein is a Ku70-Ku80 dimer involved in NHJE-mediated DNA repair [65]. To circumvent potential artifacts associated with Ku mutant parental strains that complicate mutant phenotypic characterization, Arras and colleagues reported the use of Ku protein inhibitors that improves HDR [66].

## 5. Application of CRISPR-Cas9 Technology

Despite the development of the various transformation techniques described here and their successes advancing our understanding of *Cryptococcus* species and the diseases they cause, conventional transformation methods remain time-consuming and cumbersome. Inconsistent efficiency, infrequent HDR-mediated recombination, and lack of additional selectable markers all contributed to the technical difficulties associated with continued functional genetic studies of *Cryptococcus* [67]. The recently discovered clustered regularly interspaced short palindromic repeats (CRISPR)-CRISPR associated protein 9 (Cas9) system demonstrated in a wide variety of organisms for gene editing studies has shown promise in overcoming some of these technical difficulties and accelerating genetic studies of *Cryptococcus* and other pathogenic fungi [68].

CRISPR-Cas9 technology utilizes the type II RNA-guided endonuclease Cas9 to introduce a double-stranded break (DSB) 3 bp upstream of a protospacer adjacent motif (PAM). This endonuclease activity is directed by a single-guide RNA (sgRNA) for target specificity. The DSB can be repaired by NHEJ, resulting in insertional or deletional mutation, or HDR when donor DNA with appropriate homologous sequences is co-transformed. Precision gene editing becomes possible, provided that proper donor sequences are included. CRISPR-Cas9 can significantly improve HDR-mediated gene editing over the conventional methods [69,70].

### 5.1. CRISPR-Cas9-Mediated Gene Editing and Components Elimination

CRISPR-Cas9-mediated gene editing was first demonstrated in *C. deneoformans* by Wang and colleagues in 2016, where they expressed a human codon-optimized *CAS9* gene driven by an endogenous actin promoter and a sgRNA fused with the native type III RNA polymerase promoter U6 [38]. Lacking access to a biolistic transformation apparatus, they used the electroporation method to target the *ADE2* gene as a proof of principle. A high percentage (82–88%) of NHEJ *ade2* mutants was found. When a donor DNA was present, 8 out of 20 mutant strains were found to undergo HDR-mediated knockout. To reduce possible side effects from the constitutively expressed Cas9 and sgRNA, Wang and colleagues designed a self-elimination system that allows for the excision of the CRISPR-Cas9 components from the genome following successful gene editing [38].

### 5.2. Biolistic Transformation with Self-Cleaving Ribozyme-Fused sgRNAs and Electroporation with a Transient Expression System

In the second application, Arras and colleagues expressed Cas9 and sgRNA, both driven by the native type II RNA polymerase promoters, in a *C. neoformans* (*C. neoformans var. grubii*) strain using biolistic transformation. The inclusion of two self-cleaving ribozymes (Hammerhead and Hepatitis Delta Virus (HDV) ribozymes) ensures that sgRNAs remain unmodified [66]. The inclusion of a donor DNA resulted in 70 HDR-mediated gene disruption mutants following transformation [66]. Fan and colleagues provided further improvements through a transient Cas9 expression system (TRACE) that can be delivered through electroporation [39,41].

### 5.3. Transformation with Single Vectors Expressing Cas9 and sgRNA and a Cas9-sgRNA Ribonucleoprotein (RNP) Complex

Finally, we reported two additional approaches for CRISPR-Cas9-mediated gene editing in *C. neoformans* and *C. deneoformans* by electroporation and biolistic transformation. A single plasmid vector expressing both the Cas9 and a gene-specific sgRNA was adopted in the first approach. The inclusion of two Bsm Bl restriction sites for the insertion of sgRNAs into the plasmid improves efficiency in positive clone selection [40]. The plasmid DNA can be delivered by electroporation and biolistic transformation. In the second approach, the CRISPR-Cas9 components are delivered as a ribonucleoprotein (RNP) complex through electroporation. In this application, the commercially sourced recombinant Cas9 enzyme and chemically synthesized sgRNAs were mixed and delivered via electroporation [40]. This approach reduces the intense work involved in constructing expression vectors and eliminates concerns over any activities that residual Cas9 or sgRNA may have but is generally considered to be technically more challenging. For the electroporation of *C. deneoformans*, limits on the ionic strength of the RNP reaction buffer (150 mM KCl) may restrict the amount of RNP being mixed with the cells to avoid arching. This constraint may contribute to a relatively lower number of transformants obtained compared to the traditional DNA based approach [40]. The delivery of RNPs via biolistic transformation is possible in plant research [71], but it has yet to be tested in *Cryptococcus* or other fungi. Table 2 summarizes the major CRISPR-Cas9 applications utilized in *Cryptococcus* studies.

## 6. Conclusions

The recent advances in the development of different transformation methods have revolutionized various areas of cryptococcal research. Such advances have helped to propel the field from an under-represented area of investigation to one that is at the forefront of medical mycology research. The development of a novel transformation approach without relying on the costly electroporator or biolistic apparatus would further facilitate genetic studies of this important pathogen. The development of liposome-mediated transformation method without the requirement of generating protoplasts may offer an advantageous edge in this regard.

## Figures and Tables

**Table 1:** Current transformation methods for *Cryptococcus* species.

Method	Key features	References
Electroporation	Easy, both time constant and exponential decay pulse types, episomal DNA maintenance.	[20,37,42]
Biolistic transformation	Versatile and efficient, preferred for gene knockout, high initial cost.	[43,51]
*Agrobacterium tumefaciens*-mediated transformation (ATMT)	Feasible for insertional mutagenesis, require co-cultures, not for gene knockout.	[44,52]
PEG/CaCl2-mediated protoplast, REMI, and liposome-mediated transformation	Not reported	

**Table 2:** CRISPR-Cas9 approaches for *Cryptococcus* species.

gRNA Expression	Delivery Method	Species	References
RNA Pol III promoter (U6)	Electroporation Biolistic transformation	*C. denewformans* *C. newformans*	[38,39,40,41]
RNA Pol II promoter with self-cleaving ribozymes	Biolistic transformation	*C. newformans*	[66]
Ribonucleotide protein (RNP) complex	Electroporation	*C. denewformans*	[40]

## Data Availability

This review did not report any unpublished studies.
